# Routine Life-Course Health Records in Infancy Predict Being Overweight in Childhood and Adolescence: The TMM BirThree Cohort Study

**DOI:** 10.3390/children13030334

**Published:** 2026-02-26

**Authors:** Genki Shinoda, Mami Ishikuro, Taeka Matsubara, Aoi Noda, Keiko Murakami, Masatsugu Orui, Hirohito Metoki, Masahiro Kikuya, Atsushi Hozawa, Shinichi Kuriyama, Kenji Nakamura, Taku Obara

**Affiliations:** 1 Tohoku Medical Megabank Organization, Tohoku University, 2-1 Seiryo-machi, Aoba, Sendai 980-8573, Japan; mami.ishikuro.e5@tohoku.ac.jp (M.I.); aoi.noda.b4@tohoku.ac.jp (A.N.); keiko.murakami.d6@tohoku.ac.jp (K.M.); masatsugu.orui.e5@tohoku.ac.jp (M.O.); hmetoki@tohoku-mpu.ac.jp (H.M.); kikuyam@med.teikyo-u.ac.jp (M.K.); atsushi.hozawa.a6@tohoku.ac.jp (A.H.); shinichi.kuriyama.e6@tohoku.ac.jp (S.K.); taku.obara.a5@tohoku.ac.jp (T.O.); 2 Graduate School of Medicine, Tohoku University, 2-1 Seiryo-machi, Aoba, Sendai 980-8573, Japan; 3 Interfaculty Initiative in Information Studies, The University of Tokyo, 7-3-1 Hongo, Bunkyo, Tokyo 113-0033, Japan; q-tmatsubara@g.ecc.u-tokyo.ac.jp; 4 Graduate School of Medicine, The University of Tokyo, 7-3-1 Hongo, Bunkyo-ku, Tokyo 113-0033, Japan; 5 International Research Institute of Disaster Science, Tohoku University, 468-1 Aoba, Aramaki, Aoba-ku, Sendai 980-8572, Japan; 6 Faculty of Medicine, Tohoku Medical and Pharmaceutical University, 1-15-1 Fukumuro, Miyagino-Ku, Sendai 983-8536, Japan; 7 Department of Hygiene and Public Health, School of Medicine, Teikyo University, 2-11-1, Kaga, Itabashi-ku, Tokyo 173-8605, Japan; 8 Center for Mathematics and Data Science, Gunma University, 4-2 Aramaki-machi, Maebashi 371-8510, Japan; nac-k@gunma-u.ac.jp

**Keywords:** childhood overweight, life-course data, predictive model, body mass index, obesity

## Abstract

**Highlights:**

**What are the main findings?**

- Routine life-course health records up to 18–23 months were used to predict overweight risk.
- Overweight risk could be predicted from early childhood through adolescence.
- Predictive performance was moderate to high in early and middle childhood, but modest in adolescence.

**What are the implications of the main findings?**

- Early-life health records can help identify children at high risk of overweight.
- The model demonstrates feasibility for real-world clinical and public health implementation.

**Abstract:**

**Background/Objectives**: Being overweight in childhood is a strong predictor of later obesity and related health disorders, underscoring the importance of the early identification of at-risk children. The aim of this study was to develop a prediction model for being overweight across childhood and adolescence using routine life-course health records available up to 18–23 months of age. **Methods**: We analyzed 1581 participants from the Tohoku Medical Megabank Birth and Three-Generation Cohort Study and constructed multivariable logistic regression models to predict being overweight at 36–47 months, 6 years, 11 years, and 14 years. Predictors included being overweight at 18–23 months, maternal characteristics, birth weight, and body mass index changes from birth to 18–23 months. Model performance was evaluated using the area under the curve (AUC), calibration, Brier scores, risk by score range, and stratified 10-fold cross-validation to assess the stability and robustness of predictive performance. **Results**: Being overweight at 18–23 months consistently showed strong associations with later overweight status. Model discrimination was moderate to high for early and middle childhood (AUC 0.873 at 36–47 months; 0.772 at 6 years) but modest for adolescence (AUC 0.720 and 0.692 at 11 and 14 years). Cross-validation demonstrated stable predictive performance across all age groups. Calibration and overall predictive accuracies were acceptable across all age groups. **Conclusions**: These results indicate that routine early life-course health records moderately predict the risk of being overweight, supporting their practical potential for early preventive interventions.

## 1. Introduction

Childhood obesity increases the risk of various comorbidities, including type 2 diabetes, cardiovascular disease, and metabolic syndrome [1,2]. Moreover, childhood obesity tends to persist in adulthood, with approximately 55% of school-aged children and 80% of adolescents becoming adults with obesity [3]. This “tracking” phenomenon is associated with increased risks of adult-onset metabolic abnormalities [1], cardiovascular diseases [4], and even higher mortality rates [5]. Because obesity is notoriously difficult to reverse once established, preventive measures beginning in childhood are of paramount importance [6].

Being overweight, which is a stage preceding obesity, increases the risk of adult obesity [7,8,9]. For instance, children who are overweight at 2 years of age have an approximately 2.7-fold increased relative risk of adult obesity, which increases to approximately 3.6-fold at 11 years of age [7,10]. Furthermore, adolescents aged 15–17 years old who are overweight exhibit an odds ratio of approximately 22 for being overweight or obese during adulthood [10]. In addition to increasing the risk of future obesity, overweight status in childhood is associated with a higher likelihood of developing type 2 diabetes [11], cardiovascular diseases [12,13], and mental health disorders such as depression [14]. Therefore, the early identification of children at a high risk of being overweight is a critical health challenge for preventing future health consequences.

In recent years, growing attention has been paid to the use of routine health records collected throughout life, such as birth records, maternal and child health handbooks, infant health checkups, and school health checkups, to construct early predictive models [15]. Routine life-course health records possess several advantages over research-specific data, including comprehensive population coverage, temporal consistency, scalability, cost-efficiency, and feasibility for integration into existing health and public health systems. Because these data are routinely collected as part of standard care and surveillance activities, prediction models based on such records can be implemented without additional data collection burden or substantial extra costs, making them particularly suitable for real-world clinical and public health applications [15,16]. Importantly, focusing on routinely collected health records enables the development of prediction models that are inherently scalable and readily translatable into existing child health surveillance systems, rather than being confined to research settings.

The “tracking” phenomenon is observed from early infancy; children classified as overweight at approximately 18–23 months of age have a substantially higher risk of obesity later in life [17,18]. However, the strength of weight tracking from this early age is weaker than that observed in later childhood and should be interpreted as probabilistic rather than deterministic. These findings highlight the potential value of predicting long-term overweight risk at an early stage in life, such as at 18–23 months of age. These results underscore the critical importance of predicting the long-term risk of being overweight at an early stage in life, such as at 18 months of age. However, studies that predict adolescent overweight status based solely on routine life-course health records available up to 18 months of age remain extremely limited. For instance, Morandi et al. used only perinatal risk factors to predict being overweight at the age of 16 years and reported an AUC of only 0.71 [19], indicating the need for further research to support practical applications.

Regarding predictive modeling strategies, recent studies have increasingly adopted machine-learning-based approaches and have demonstrated moderate-to-good performance for childhood overweight and obesity prediction [20,21,22]. Nevertheless, most of these studies have focused on short- to mid-term outcomes or have incorporated information obtained after early infancy. As a result, evidence for long-term prediction into adolescence based solely on routinely collected data available by early childhood remains limited.

The aim of this study was to develop a prediction model for the risk of being overweight at multiple time points, from early childhood through adolescence, using routine life-course health records available up to 18–23 months of age, including data from the Maternal and Child Health Handbook, infant health checkups, and school health checkups, and evaluate its practical applicability. The novelty of this study lies in the combination of the exclusive use of routinely collected early-life health data and a multi-age prediction framework spanning early childhood through adolescence. We demonstrate that such routinely collected early-life data can enable robust and practically implementable long-term overweight risk prediction.

## 2. Materials and Methods

### 2.1. Study Design

Data were obtained from the Tohoku Medical Megabank Project Birth and Three-Generation Cohort Study (TMM BirThree Cohort Study) as previously described [23,24,25]. Pregnant women were recruited from obstetric clinics and hospitals in Miyagi Prefecture, Japan, between 2013 and 2017, and their children constituted the primary participants of the present study. School check-up data for participants in this cohort were collected from 2018 to 2024 in collaboration with the municipal boards of education and schools. Children whose parents provided proxy consent for the collection of regular school health checkup data were included. School health checkup data were integrated with infant health checkup data obtained from municipal maternal and child health departments, as well as with maternal and child health handbooks provided by parents or guardians. Of the 9459 children enrolled in the TMM BirThree Cohort, 7878 children were excluded for the following reasons: 198 participants withdrew consent; 7655 participants had missing anthropometric data (height and weight) at one or more of the following time points: birth, 18 months, 36–47 months, 6 years, 11 years, and 14 years; 25 participants had extreme body mass index (BMI) values exceeding ±5 standard deviations (SD) at one or more of the following time points: birth, 18 months, 36–47 months, 6 years, 11 years, or 14 years. The final analytical sample therefore included 1581 children. To assess potential selection bias resulting from exclusions, baseline characteristics of included and excluded participants were compared using the available demographic and perinatal data.

### 2.2. Definition of Body Type

Body type was based on sex, height, body weight, measurement date, date of birth, and gestational age. We used data from six time points: birth, 18–23 months, 36–47 months, 6 years, 11 years, and 14 years. After birth, BMI z-scores were calculated using age- and sex-specific Japanese reference values [26]. Weight status was categorized as follows: overweight, BMI z-score > 1; normal weight, BMI z-score between −2 and 1; and thin, BMI z-score < −2.

### 2.3. Statistical Analysis

Characteristics were compared between overweight and non-overweight children at 18 months of age using Welch’s *t*-test for continuous variables and the chi-square test for categorical variables.

Multivariate logistic regression was used to develop prediction models for being overweight at 36–47 months, 6 years, 11 years, and 14 years. For each age outcome, a separate regression model was fitted using variables obtained from routine life-course health records up to 18–23 months. We intentionally used conventional logistic regression models to prioritize interpretability and to reduce the risk of overfitting that may occur with more complex machine-learning approaches, particularly in moderate-sized datasets. The basic model (Model 1) included the following predictors: overweight at 18–23 months of age, child’s sex, maternal age at delivery, parity (primipara vs. multipara), maternal BMI, and maternal smoking and drinking status at the time of pregnancy recognition. These variables were selected based on our previously published work [17]. The extended model (Model 2) included all variables in Model 1, in addition to birth weight and changes in BMI from birth to 18–23 months of age. These variables were consistently reported as strong early-life predictors of later overweight [27,28,29,30]. All predictors were selected based on prior literature and our previous work, restricted to routinely collected health records, and included for prediction rather than causal inference.

Missing data were handled using multiple imputations by chained equations. Imputation was performed prior to model development, and all variables included in the analyses were used in the imputation models. Estimates were pooled across imputed datasets according to Rubin’s rules. For both models, data were divided into training (70%) and validation (30%) datasets.

The performance of Model 2 was evaluated in terms of discrimination, calibration, and clinical utility. Discrimination was assessed using the area under the receiver operating characteristic curve (AUC). To assess the stability and robustness of model performance and to reduce the risk of optimism bias associated with a single training–validation split, we additionally performed stratified 10-fold cross-validation. In this procedure, the dataset was randomly divided into 10 mutually exclusive subsets while preserving the proportion of overweight individuals. The model was trained on nine subsets and evaluated on the remaining subset, and this process was repeated 10 times so that each subset served as the validation set once. The mean AUC, SD, and corresponding 95% confidence intervals were calculated across the folds. To evaluate the stability and appropriateness of the model specifications, multicollinearity among predictors was assessed using variance inflation factors (VIF). Non-linearity of continuous predictors was explored by including quadratic terms, particularly for BMI change from birth to 18–23 months. These analyses were performed for each outcome age.

Calibration was performed using calibration plots and quantified using Brier scores. The Brier score is defined as the mean-squared difference between predicted probabilities and observed binary outcomes, with lower values indicating better predictive accuracy. To assess potential usefulness for risk stratification, predicted probabilities were categorized into score ranges and the corresponding observed overweight proportions were compared within each category (“risk by score range”).

All statistical analyses were performed using R, software (version 4.0.2, R Foundation for Statistical Computing, Vienna, Austria).

### 2.4. Ethical Approval and Consent to Participate

The TMM BirThree Cohort Study protocol was reviewed and approved by the Ethics Committee of Tohoku University Tohoku Medical Megabank Organization (approval nos. 2012-4-617, 2013-1-103-1, and 2023-4-117). This study followed the STROBE guidelines. All methods were carried out in accordance with the Declaration of Helsinki. All participants provided their informed consent at enrollment. For participants with insufficient ability to understand the study protocol at any age, with the Ethics Committee’s approval, informed consent was obtained from their guardians.

### 2.5. Declaration of the Use of AI

During the preparation of this manuscript/study, the authors used the artificial intelligence tool GPT-4o, developed by OpenAI (San Francisco, CA, USA), for the purposes of the initial drafting and editing of this manuscript. This tool provides suggestions for sentence structure and grammatical corrections. All intellectual contributions and final edits were made by the authors. The authors have reviewed and edited the output and take full responsibility for the content of this publication.

## 3. Results

### 3.1. Characteristics of the Study Participants

Participant characteristics are presented in Table 1. Overall, 327 (20.7%) children were overweight at 18–23 months old. At 18–23 months, children who were overweight had higher mean body weight at birth, greater increases in BMI from birth to 18–23 months, and higher BMI at 18–23 months than those with normal weight. Moreover, their mothers were slightly younger, had higher BMIs at pregnancy recognition, and were more likely to be multiparous. The prevalence of being overweight remained consistently higher in the overweight group than in the normal group across later ages, including 36–47 months, 6 years, 11 years, and 14 years (Table 1). Baseline characteristics of included and excluded participants are shown in Supplementary Table S1. The participants included were older than excluded participants, reflecting differences in data availability. Although some statistically significant differences were observed, the magnitude of these differences was small, and most baseline characteristics were broadly similar between the two groups.

### 3.2. Predictors of Being Overweight Across Childhood in the Extended Model

**Figure 1:**
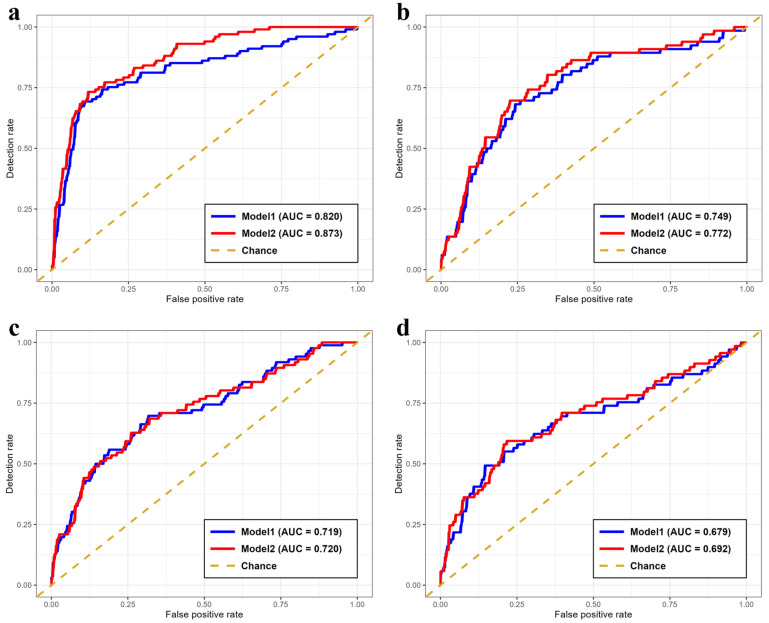
Receiver operating characteristic (ROC) curves for the prediction of being overweight. Routine life-course health records were used as predictors in both models. Predictor variables included overweight status at 18–23 months of age, child’s sex, maternal age at delivery, parity (primipara vs. multipara), maternal BMI, and maternal smoking and drinking status at the time of pregnancy recognition. Model 2 included all variables in Model 1, with the addition of birth weight and change in BMI from birth to 18–23 months. (**a**) ROC curves comparing predictive models for being overweight at 36–47 months of age. The areas under the curve (AUCs) for Models 1 and 2 were 0.820 and 0.873, respectively. (**b**) ROC curves comparing predictive models for being overweight at 6 years of age. The AUCs were 0.749 and 0.772, respectively. (**c**) ROC curves comparing predictive models for being overweight at 11 years of age. The AUCs were 0.725 and 0.726, respectively. (**d**) ROC curves comparing predictive models for being overweight at 14 years of age. The AUCs were 0.691 and 0.702, respectively.

Following the predictive performance of the basic model (Model 1) shown in Figure 1, we examined which variables contributed to the prediction of overweight across childhood using the extended model (Model 2).

In Model 2, being overweight at 18–23 months (coefficient: 1.51; 95% CI: 1.060–1.971), smoking at pregnancy recognition (coefficient: 0.92; 95% CI: 0.313–1.508), body weight at birth (coefficient: 1.82; 95% CI: 1.226–2.438), and change in BMI from birth to 18–23 months (coefficient: 0.54; 95% CI: 0.364–0.732) were retained as predictors contributing to the predicted probability of overweight at 36–47 months (Table 2). At 6 years of age, being overweight at 18–23 months (coefficient: 1.06; 95% CI: 0.535–1.581) and maternal BMI at pregnancy recognition (coefficient: 0.23; 95% CI: 0.172–0.294) were retained as predictors in the model (Table 2). Similarly, at 11 years of age, being overweight at 18–23 months (coefficient: 0.64; 95% CI: 0.158–1.124) and maternal BMI at pregnancy recognition (coefficient: 0.20; 95% CI: 0.145–0.259) contributed to the predicted probability of overweight (Table 2). For overweight at 14 years of age, being overweight at 18–23 months (coefficient: 0.94; 95% CI: 0.440–1.437), sex (male) (coefficient: −0.47; 95% CI: −0.841 to −0.108), and body weight at birth (coefficient: 0.59; 95% CI: 0.018–1.163) were retained as predictors (Table 2).

Figure 1 shows the ROC curves for predicting being overweight at 36–47 months, 6 years, 11 years, and 14 years of age (panels a–d). The AUCs in Model 1 were 0.820, 0.749, 0.719, and 0.679 for ages 36–47, 6, 11, and 14 years, respectively, whereas those in Model 2 were 0.873, 0.772, 0.720, and 0.692, respectively. To assess the stability and robustness of model performance, stratified 10-fold cross-validation was additionally performed. The cross-validation results demonstrated stable predictive performance across different data splits for all prediction ages. The detailed cross-validation results are presented in Table 3. Multicollinearity among predictors was minimal across all models, with variance inflation factors ranging from 1.04 to 2.32, indicating no evidence of problematic collinearity. Exploration of non-linearity using quadratic terms for BMI change from birth to 18–23 months did not reveal statistically significant non-linear associations at any outcome age (*p* = 0.247 at 36–47 months, *p* = 0.128 at 6 years, *p* = 0.180 at 11 years, and *p* = 0.409 at 14 years), supporting the appropriateness of linear model specifications.

### 3.3. Calibration and Overall Predictive Performance of the Model 2

**Figure 2:**
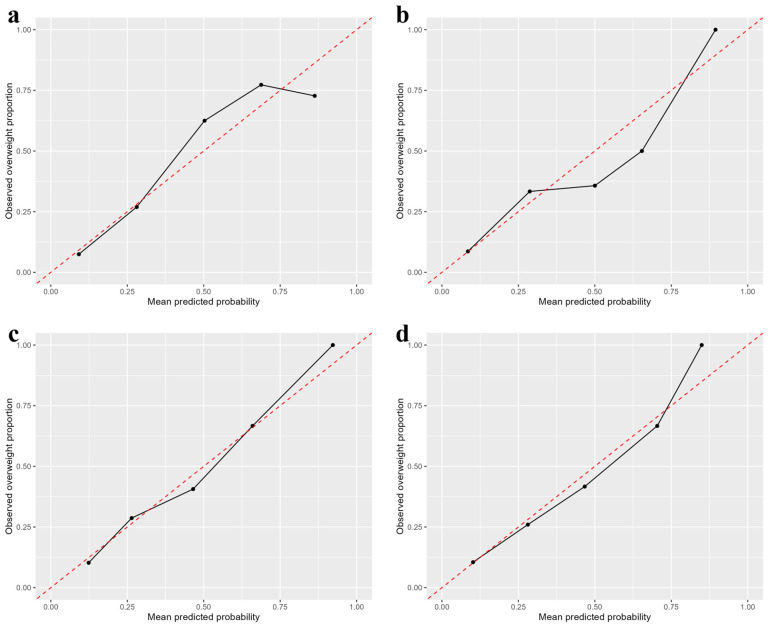
Calibration plots for Model 2 predicting being overweight. Calibration plots assessing the agreement between the predicted probability of being overweight and observed proportion of overweight individuals across the five predicted risk groups. Predictions were generated using Model 2, which included overweight status at 18–23 months of age, child’s sex, maternal age at delivery, parity (primipara vs. multipara), maternal BMI, maternal smoking and drinking status at the time of pregnancy recognition, birth weight, and changes in BMI from birth to 18–23 months. The red dashed line represents a perfect calibration, where the predicted and observed risks are equal. Points above the line indicate an overestimation, whereas points below indicate an underestimation. (**a**) Calibration plot for predicting being overweight at 36–47 months of age. (**b**) Calibration plot for predicting being overweight at 6 years of age. (**c**) Calibration plot for predicting being overweight at 11 years of age. (**d**) Calibration plot for predicting being overweight at 14 years of age.

The calibration plots for Model 2 are presented in Figure 2. Across all age groups, the predicted probabilities were in good agreement with the observed proportions of overweight individuals, particularly in the lower-to-middle predicted probability ranges. However, the discrepancy between predicted and observed risks increased at higher predicted probabilities. Consistent with these visual assessments, the Brier scores indicated good overall predictive accuracy: 0.107 for 36–47 months, 0.105 for 6 years, 0.130 for 11 years, and 0.111 for 14 years of age. These values reflected a low prediction error and supported the acceptable calibration of the model across childhood.

**Figure 3:**
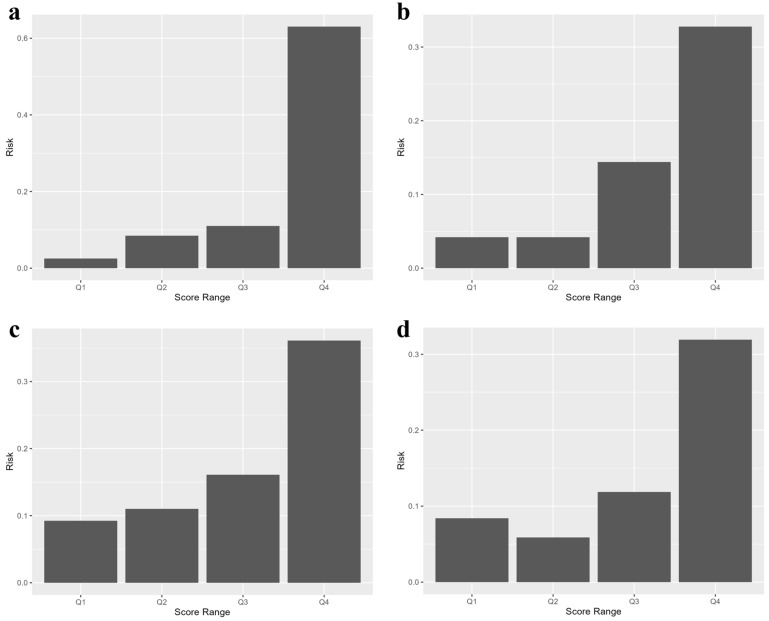
Risk by score range for being overweight across childhood. Panels (**a**–**d**) show the proportion of children classified as overweight within each quartile of the predicted risk score for the four prediction models. In all models, the risk of being overweight progressively increased with higher score quartiles. Children in the highest quartile (Q4) consistently demonstrated an elevated risk compared with those in the lower quartiles (Q1–Q3), indicating good discrimination of the risk score. Although the magnitude of risk varied across the models, the overall pattern remained similar, with Q4 showing the steepest increase in predicted risk.

Figure 3 illustrates the observed risk of being overweight across score ranges (quartiles of the predicted risk score) for each age group. In all age categories (panels a–d), the risk of being overweight increased progressively with higher score ranges, indicating that the model effectively stratified children into distinct risk groups. The highest score range (Q4) consistently demonstrated a substantially elevated risk of being overweight compared with the lower ranges, with risk levels rising sharply in Q4 relative to those in Q1–Q3. Thus, the prediction model had good risk stratification ability across childhood.

## 4. Discussion

We developed a prediction model for the risk of being overweight in childhood using routine life-course health records obtained up to 18–23 months of age. The model could predict being overweight at multiple time points throughout childhood with moderate-to-high accuracy. In particular, the prediction of the risk of being overweight at 3 and 6 years of age was improved by incorporating birth weight and changes in BMI from birth to 18–23 months. In contrast, the predictive accuracy for being overweight at 11 and 14 years of age showed little improvement. The limited improvement in predictive accuracy for overweight at 11 and 14 years of age likely reflects fundamental differences in the determinants of adiposity between early childhood and adolescence. Our results suggest that this model has the potential for clinical and public health applications.

In this study, growth patterns observed until 18–23 months of age played a major role in determining the risk of being overweight later in childhood. Overweight status at 18–23 months was a consistently strong predictor of being overweight throughout preschool age (36–47 months of age) and adolescence (14 years). The importance of identifying overweight individuals at this age has also been highlighted in previous studies [17,31]. Additionally, birth weight and changes in BMI during infancy substantially contributed to the predictive accuracy of being overweight in the preschool period. The role of birth weight as a risk factor for being overweight or obese in preschool has been supported by multiple studies, with reported odds ratios of 2–3 [32,33,34]. Furthermore, accelerated weight gain or BMI during the first 0–24 months of life is positively associated with being overweight or obese later [29,30,35,36]. In contrast, the effect of these early life factors was diminished by adolescence and improvements in model performance were limited at older ages. Birth weight has little predictive value for adiposity in adolescence [37,38] and associations between weight gain during early childhood (ages 2–7 years) and being overweight later remain largely unchanged even after adjustments for birth weight [39]. Although increases in weight or BMI during infancy are associated with dual energy X-ray absorptiometry (DXA)-measured body fat at 13–14 years of age [37] and confer a three- to four-fold increased risk of being overweight or obese from childhood into adolescence [40], our results did not show a significant association between early BMI gain and being overweight in adolescence.

We comprehensively evaluated the performance of the prediction model across multiple dimensions, including the AUC, calibration (calibration plots), and overall predictive accuracy (Brier score). These assessments demonstrated that the model achieves a level of performance that is potentially applicable in clinical settings. In particular, the prediction of the risk of being overweight at 36–47 months, 6 years, and 11 years demonstrated adequate discriminatory ability, with AUC values exceeding 0.7 [19,41]. In contrast, the predictive performance for overweight adolescents was modest. This may reflect the absence of key long-term determinants of being overweight and adiposity in the prediction model. Adiposity development during adolescence is influenced by a complex interplay of biological maturation, hormonal changes, lifestyle behaviors, psychosocial factors, and environmental exposures, many of which are not captured by routine health records obtained in early infancy [3,42,43,44,45,46]. Therefore, the modest predictive performance observed for adolescent outcomes in the present study should be interpreted cautiously and does not imply that early-life data alone are sufficient to fully explain overweight development during this later developmental stage. In addition, it should be noted that calibration tended to be poorer at higher predicted risk levels. This pattern may be partly attributable to the relatively small number of individuals in the highest-risk categories and increased heterogeneity within this subgroup. In addition, unmeasured factors that become more relevant later in childhood, such as lifestyle behaviors, family environment, and socioeconomic conditions, may partly contribute to miscalibration among high-risk individuals [12,13,19,47]. From a practical perspective, this potential overestimation suggests that predicted probabilities in the highest risk range should be interpreted as indicators of relative risk rather than precise absolute risk, and may be most useful for identifying children who could benefit from further assessment or preventive support [41].

This study had several important strengths. To the best of our knowledge, this is the first study to demonstrate that the risk of being overweight from early childhood through adolescence can be predicted using only routinely collected public health data available at 18–23 months of age [33,47]. The predictive performance observed in this study was comparable to that reported in recent machine-learning-based approaches for childhood overweight prediction, particularly for short- to mid-term outcomes [15,16,48]. The ability to generate moderate predictive accuracy across multiple developmental stages highlights the potential feasibility of implementing these models in real-world clinical and public health settings [18]. As the required variables are routinely collected during standard health checkups in early childhood, the model can be incorporated into existing maternal and child health surveillance systems without additional burden on families or healthcare providers [49]. Ultimately, the early identification of children at an elevated risk of being overweight may enable timely preventive interventions that mitigate long-term consequences throughout the life course [18,50]. Nevertheless, predictive performance for adolescent overweight was only moderate; therefore, predictions for this age group should be interpreted cautiously and regarded primarily as exploratory or supportive information rather than definitive individual-level risk estimates.

The present study had some limitations. First, the most important limitation of the present study is the lack of external validation. The prediction models were developed and evaluated using a single Japanese cohort, and external validation was not performed. Therefore, the generalizability of the findings to other populations, countries, and healthcare systems remains uncertain, and independent validation in diverse settings is warranted before broader implementation [41]. Second, BMI does not fully reflect body fat mass; therefore, it may not precisely capture adiposity-related risks during childhood and adolescence [51,52]. Although BMI is widely used in epidemiological research and clinical practice, direct measures of adiposity, such as DXA and bioelectrical impedance analysis, were not available in this study. Third, although we performed stratified 10-fold cross-validation to assess the stability and robustness of model performance, external validation using an independent cohort was not conducted. Cross-validation provides an internal assessment of model stability but does not fully address the generalizability of the models to different populations or settings. Therefore, external validation in independent cohorts remains necessary to confirm the reproducibility and generalizability of the findings. Fourth, several factors known to influence childhood growth and adiposity were not included in the present models because the prediction models were intentionally based on routinely collected data available up to 18–23 months of age. Although this design enhances feasibility and scalability for real-world public health implementation, the absence of these variables may have limited predictive performance, particularly in later childhood and adolescence. Finally, anthropometric measurements were obtained from routine health checkups conducted at multiple facilities, and some degree of measurement error is unavoidable, particularly in infancy. Although age- and sex-standardized BMI z-scores were used and implausible extreme values were excluded, residual measurement error may remain.

## 5. Conclusions

This study demonstrates that routine life-course health records collected at 18–23 months of age can be used to develop risk prediction models for childhood overweight with reasonable accuracy throughout early and middle childhood. Although the predictive performance declined in adolescence, the results suggest that early life growth indicators contribute to the predictive performance of models aimed at identifying children at elevated long-term risk of overweight. The applicability of these models for adolescent and long-term prediction should therefore be interpreted cautiously. Further external validation in independent populations is warranted, and these prediction models may be useful for early risk stratification in clinical or public health settings, such as routine child health checkups.

## Figures and Tables

**Table 1:** Baseline characteristics of the study participants.

		Overall	Normal Weight at 18–23 Months	Overweight at 18–23 Months	*p*-Value
n		1581	1254	327	
Characteristics of children					
Sex, n (%)	Female	769 (48.6)	596 (47.5)	173 (52.9)	0.095
	Male	812 (51.4)	658 (52.5)	154 (47.1)	<0.001
Body weight at birth (g)		3072.2 (388.1)	3046.8 (392.6)	3169.7 (354.5)	<0.001
Change in BMI from birth to 18–23 months (kg/m^2^)		3.8 (1.5)	3.4 (1.3)	5.2 (1.3)	<0.001
BMI at 18–23 months of age (kg/m^2^)		16.3 (1.2)	15.8 (0.8)	18.0 (0.9)	<0.001
Overweight at 36–47 months of age, n (%)	Yes	336 (21.3)	132 (10.5)	204 (62.4)	<0.001
	No	1245 (78.7)	1122 (89.5)	123 (37.6)	
Overweight at 6 years of age, n (%)	Yes	219 (13.9)	118 (9.4)	101 (30.9)	<0.001
	No	1362 (86.1)	1136 (90.6)	226 (69.1)	
Overweight at 11 years of age, n (%)	Yes	287 (18.2)	187 (14.9)	100 (30.6)	<0.001
	No	1294 (81.8)	1067 (85.1)	227 (69.4)	
Overweight at 14 years of age, n (%)	Yes	229 (14.5)	142 (11.3)	87 (26.6)	<0.001
	No	1352 (85.5)	1112 (88.7)	240 (73.4)	
Characteristics of mothers					
Maternal age at the time of childbirth (years)		26.9 (3.9)	27.0 (3.9)	26.5 (3.9)	0.030
Maternal BMI at pregnancy recognition (kg/m^2^)		20.5 (2.7)	20.3 (2.6)	21.1 (3.0)	
Smoking at pregnancy recognition, n (%)	Yes	114 (7.2)	82 (6.5)	32 (9.8)	0.057
	No	1467 (92.8)	1172 (93.5)	295 (90.2)	
Drinking alcohol at pregnancy recognition, n (%)	Yes	153 (9.7)	120 (9.6)	33 (10.1)	0.858
	No	1428 (90.3)	1134 (90.4)	294 (89.9)	
Maternal parity, n (%)	Primipara	1189 (75.2)	948 (75.6)	241 (73.7)	0.525
	Multipara	392 (24.8)	306 (24.4)	86 (26.3)	

Data are shown as the mean ± standard deviation for continuous variables and n (%) for categorical variables. Differences were evaluated using the *t*-test for continuous variables and chi-square test for categorical variables. BMI, body mass index.

**Table 2:** Prediction models for being overweight including life-course health data as predictors.

	OR	Coefficient	95% Confidence Interval
Predictive model for being overweight at 36–47 months of age			
Overweight at 18–23 months of age: Yes	**4.54**	1.51	1.060 to 1.971
Sex: Male	0.89	−0.12	−0.479 to 0.239
Maternal age at the time of childbirth (years)	1.03	0.03	−0.011 to 0.079
Maternal parity: Multipara	0.86	−0.15	−0.568 to 0.255
Maternal BMI at pregnancy recognition (kg/m^2^)	1.06	0.06	−0.002 to 0.124
Drinking alcohol at pregnancy recognition: Yes	0.81	−0.21	−0.852 to 0.394
Smoking at pregnancy recognition: Yes	**2.50**	0.92	0.313 to 1.508
Body weight at birth (g)	**6.19**	1.82	1.226 to 2.438
Change in BMI from birth to 18–23 months (kg/m^2^)	**1.72**	0.54	0.364 to 0.732
Predictive model for being overweight at 6 years of age			
Overweight at 18–23 months of age: Yes	**2.88**	1.06	0.535 to 1.581
Sex: Male	1.35	0.30	−0.085 to 0.690
Maternal age at the time of childbirth (years)	0.98	−0.02	−0.065 to 0.030
Maternal parity: Multipara	0.76	−0.28	−0.743 to 0.161
Maternal BMI at pregnancy recognition (kg/m^2^)	**1.26**	0.23	0.172 to 0.294
Drinking alcohol at pregnancy recognition: Yes	1.64	0.50	−0.119 to 1.070
Smoking at pregnancy recognition: Yes	1.01	0.01	−0.728 to 0.689
Body weight at birth (g)	1.77	0.57	−0.041 to 1.185
Change in BMI from birth to 18–23 months (kg/m^2^)	1.11	0.11	−0.065 to 0.280
Predictive model for being overweight at 11 years of age			
Overweight at 18–23 months of age: Yes	**1.90**	0.64	0.158 to 1.124
Sex: Male	1.14	0.13	−0.206 to 0.468
Maternal age at the time of childbirth (years)	0.99	−0.01	−0.055 to 0.029
Maternal parity: Multipara	0.96	−0.04	−0.432 to 0.343
Maternal BMI at pregnancy recognition (kg/m^2^)	**1.22**	0.20	0.145 to 0.259
Drinking alcohol at pregnancy recognition: Yes	**1.82**	0.60	0.059 to 1.114
Smoking at pregnancy recognition: Yes	1.01	0.01	−0.623 to 0.598
Body weight at birth (g)	1.32	0.28	−0.252 to 0.816
Change in BMI from birth to 18–23 months (kg/m^2^)	1.10	0.10	−0.059 to 0.255
Predictive model for being overweight at 14 years of age			
Overweight at 18–23 months of age: Yes	**2.56**	0.94	0.440 to 1.437
Sex: Male	**0.62**	−0.47	−0.841 to −0.108
Maternal age at the time of childbirth (years)	0.99	−0.01	−0.053 to 0.040
Maternal parity: Multipara	0.83	−0.18	−0.615 to 0.232
Maternal BMI at pregnancy recognition (kg/m^2^)	**1.18**	0.16	0.102 to 0.224
Drinking alcohol at pregnancy recognition: Yes	0.96	−0.04	−0.706 to 0.569
Smoking at pregnancy recognition: Yes	1.39	0.33	−0.322 to 0.944
Body weight at birth (g)	**1.80**	0.59	0.018 to 1.163
Change in BMI from birth to 18–23 months (kg/m^2^)	1.01	0.01	−0.152 to 0.174

OR, odds ratio; BMI, body mass index. Statistically significant odds ratios (95% confidence intervals not crossing 1.0) are shown in bold.

**Table 3:** Predictive performance assessed using stratified 10-fold cross-validation.

Outcome Age	Mean AUC	SD	95% CI
36–47 months of age	0.835	0.03	0.798–0.873
6 years of age	0.757	0.051	0.693–0.821
11 years of age	0.702	0.016	0.682–0.723
14 years of age	0.697	0.059	0.624–0.769

AUC, area under the curve; SD, standard deviation; CI, confidence interval.

## Data Availability

The datasets generated and/or analyzed in the current study are available from the corresponding author upon reasonable request.

## Supplementary Materials

The following supporting information can be downloaded at: https://www.mdpi.com/article/10.3390/children13030334/s1, Table S1: Comparison of baseline characteristics between included and excluded participants.

## Abbreviations

The following abbreviations are used in this manuscript:

AUC	area under the curve
BMI	body mass index
CI	confidence interval
DXA	dual energy X-ray absorptiometry
OR	Odds ratio
ROC	receiver operating characteristic
RR	relative risk
SD	standard deviation
