# Supplementary material for: Routine Life-Course Health Records in Infancy Predict Being Overweight in Childhood and Adolescence: The TMM BirThree Cohort Study
Source: Children (Basel). 2026 Feb 26;13(3):334. doi: 10.3390/children13030334 (PMC13025306; doi:10.3390/children13030334)
Supplement: Supplementary file 1 [file children-13-00334-s001.zip › children-4137256-supplementary.pdf]

Supplementary table S1. Comparison of baseline characteristics between included and excluded participants

		Overall	included participants	excluded participants	*P*-value
n		9,223	1,581	7,642	
**Characteristics of children**					
Sex, n (%)	Female	4,500 (48.8)	769 (48.6)	3,731 (48.8)	0.917
	Male	4,723 (51.2)	812 (51.4)	3,911 (51.2)	
Current age (years)		14.90 (2.87)	17.94 (1.61)	14.28 (2.67)	<0.001
Body weight at birth (g)		3,049.14 (405.40)	3,073.11 (388.30)	3,043.00 (409.47)	0.009
Body height at birth (cm)		49.41 (2.30)	49.48 (2.09)	49.39 (2.35)	0.198
**Characteristics of mothers**					
Maternal age at the time of childbirth (years)		28.44 (4.44)	26.90 (3.93)	28.76 (4.47)	<0.001
Maternal BMI at pregnancy recognition (kg/m^2)		20.71 (3.83)	20.47 (2.67)	20.77 (4.07)	0.014
Smoking at pregnancy recognition, n (%)	Yes	270 (2.9)	87 (5.5)	183 (2.4)	<0.001
	No	6,166 (66.9)	1,221 (77.2)	4,945 (64.7)	
	NA	2,787 (30.2)	273 (17.3)	2,514 (32.9)	
Drinking alcohol at pregnancy recognition, n (%)	Yes	604 (6.5)	133 (8.4)	471 (6.2)	<0.001
	No	5,721 (62.0)	1148 (72.6)	4,573 (59.8)	
	NA	2,898 (31.4)	300 (19.0)	2,598 (34.0)	
Maternal parity, n (%)	Primipara	6,312 (68.4)	1,138 (72.0)	5,174 (67.7)	0.001
	Multipara	2,409 (26.1)	381 (24.1)	2,028 (26.5)	
	NA	502 (5.4)	62 (3.9)	440 (5.8)	

Data are shown as the mean ± standard deviation for continuous variables and n (%) for categorical variables. Differences between included and excluded participants were evaluated using the t-test for continuous variables and chi-square test for categorical variables after exclusion of implausible extreme values (±5 standard deviations).

BMI, body mass index; NA, Not Available.
